# The Sustainable Development Goals Psychological Inventory: A Network Analysis in Italian University Students

**DOI:** 10.3390/ijerph191710675

**Published:** 2022-08-27

**Authors:** Andrea Svicher, Alessio Gori, Annamaria Di Fabio

**Affiliations:** 1 Department of Education, Languages, Intercultures, Literatures, and Psychology (Psychology Section), University of Florence, 50135 Florence, Italy; 2 Department of Health Sciences, University of Florence, 50135 Florence, Italy

**Keywords:** sustainable development goals, sustainable development goals psychological inventory, psychology of sustainability, psychology of sustainable development, network analysis

## Abstract

The Sustainable Development Goals Psychological Inventory (SDGPI) is a recently developed self-report questionnaire that assesses interest, motivation, and self-efficacy associated with each of the 17 Sustainable Development Goals (SDGs) advanced by the United Nations. This study aims to investigate, via network analysis, (a) the relationships between interest, motivation, and self-efficacy for each SDG and (b) the most central SDGs. To this end, 417 Italian university students (73.9% females and 26.1% males; mean age: 22.20; DS = 3.02) were assessed through the SDGPI, and two network structures were estimated. The first network structure investigates links (edges) between interest, motivation, and self-efficacy in relation to each specific SDG. The second network structure investigates most central SDGs as the sum of interest, motivation, and self-efficacy for each specific SDG. Regarding results, the first network structure showed that five SDGs had strong and statistically significant edges between interest, motivation, and self-efficacy; seven SDGs had strong and statistically significant edges between interest and motivation but not self-efficacy; five SDGs had no statistically significant edges linking the other dimensions. The second network structure revealed that SDG 2 (Zero Hunger) and SDG 7 (Affordable and clean energy) were the most central nodes. Implications for research, tailor-made interventions, and prevention were discussed.

## 1. Introduction

The United Nations (UN) established the Agenda for Sustainable Development Goals (SDGs) on 25 September 2015 [1]. The agenda encloses 17 goals applicable to countries and regions all around the globe [2]. The SDGs have sustainability as the centerpiece for the development of human society and natural environments [3]. Sustainability has three pillars, namely environmentally sound decisions, economically viable decisions, and socially equitable decisions [4]. However, compared to the previous global development program, SDGs in the 2030 Agenda have their indivisibility as a unique feature [5]. Thus, the 2030 Agenda conceives the development and targets of each SDG as interdependent and interlinked [6,7]. In this line, sustainability science [8,9,10] has recently emerged as a multidisciplinary framework focused on bringing together diverse fields of study to realize the SDGs of the UN [11]. Within sustainability science, the psychology of sustainability and sustainable development [12,13,14] offers a new perspective to enrich sustainability science’s background [15]. The psychology of sustainability and sustainable development aims to study the psychological processes (e.g., thoughts and behaviors) involved in decisions regarding environmental and human society issues [14]. In this framework, the psychology of sustainability and sustainable development contributes to the three pillars of sustainability, keeping in mind the idea that psychological processes involved in all the environments (natural, personal, social, organizational, community, digital, cross-cultural, etc., up to global environments) can be managed not only by following ever-decreasing supplies of resources but also by revitalizing psychological processes from a positive point of view [16]. Accordingly, the levels of quality of life and well-being experienced in each environment, as well as in the interconnection of environments, became highly relevant [13]. From the psychology of sustainability and sustainable development points of view, it is also highly relevant to develop and implement lines of research and intervention based on a strength-based prevention perspective [17]. To this end, Di Fabio and Rosen [18] recently developed the Sustainable Development Goals Psychological Inventory (SDGPI). The SDGPI [18] was developed to evaluate individuals’ psychological self-perception of the SDGs, focusing on three relevant psychological components related to actions and performance, namely interest [19], motivation [20], and self-efficacy [21]. Interest is defined as a need or desire to pay selective attention to a certain activity, goal, or field of study that is important to the person [22]. Motivation is the psychological force that drives conduct in humans and gives them direction and purpose [22]. Self-efficacy regards the psychological representation that individuals have of their own ability to organize and carry out specific performances or activities [19]. It is conceived as a dynamic core feature of a person [21]. Several theories have examined the relationships between interest, motivation, and self-efficacy. According to the interest model [19], self-efficacy and response expectations play a role in modulating interest. Interest in a particular activity arises when people feel efficacious and expect positive results. In contrast, people show disinterest in activities in which they are unsure of their ability to be successful, or they expect negative or neutral outcomes. As a consequence, interests lead to intentions for additional activities, which raises the possibility of subsequent practice. Practice can produce successful activities, which, in turn, could increase self-efficacy in performing a certain task [19]. Self-Determination Theory (SDT) [20] is a different psychological theory that connects motivation and interest. SDT distinguishes intrinsic (innate drive to explore possibilities and new opportunities) and extrinsic motivation (stemming from external causes), as well as amotivation (a lack of motivation). Interest contributes to intrinsic motivation by starting and directing attention and exploratory behaviors [20]. Starting from these premises, self-efficacy is a crucial variable in the SGDPI framework [18]. Furthermore, the virtuous circle of interest, motivation, and self-efficacy for activities increases the likelihood of subsequent practice [18].

From this perspective, this inventory permits an in-depth evaluation of the three components (interest, motivation, and self-efficacy), allowing one to acquire more accurate data to develop successful actions related to the seventeen SDGs [23,24], tailoring the interventions in relation to specific targets and contexts. According to the authors, these tailor-made actions could better cope with peculiar needs concerning different realities, people, areas, and countries, taking advantage of refining them on the basis of findings that emerged from the SDGPI [18]. Furthermore, results carried out via the SDGPI could also foster the structuring of programs inherent to preventive perspectives and primary, secondary, and tertiary prevention [25], taking into account the observed levels of interests, motivations, and self-efficacy in relation to the seventeen SDGs [18]. Primary prevention is aimed at preventing the insurgence of a problem before it starts. Secondary prevention aims at terminating the process related to problems. Tertiary prevention refers to the prevention of complications related to problems [25]. In this framework, positive strength-based preventive perspectives [17], as well as an advanced primary prevention perspective [12,13,26,27], could be relevant, involving actions relative to the care and consideration of the natural world (connectedness to nature) [28] and people with their interaction (empathy or compassion) [17,29,30], as well as their purposes in terms of meaningfulness and self-orientation [31].

Previous results have investigated the SDGPI via the latent factor theory [18]. However, alternatively to the factor approach, the network approach has recently emerged [32,33] and has been applied in several psychological research areas, such as personality [34,35], emotions [36], empathy [37], perfectionism [38], the dark triad [39], mental disorders [40], well-being [41], meaning at work, and decent work [42,43]. According to this approach, psychological self-perception of the SDGs does not arise from latent factors but derives from the reciprocal interaction between its observable indicators (scale items) [44]. Thus, following the SDGPI measurement model, the network realm can provide a novel perspective to analyze the link between interest, motivation, and self-efficacy. From this perspective, it is possible to analyze the network structure of SDGPI as composed of nodes (item) and edges (the links between nodes). Two approaches were selected. The first approach involves a network at the item level composed of 51 nodes that are single SDG items: Interest (17 nodes), motivation (17 nodes), and self-efficacy (17 nodes). In this approach, the presence of a link (edge) among interest, motivation, and self-efficacy in relation to a specific SDG indicates that they are reciprocally related and may occur simultaneously [45,46]. The links (edges) can also occur in the following combinations: Between interest and motivation but not self-efficacy; between interest and self-efficacy but not motivation; and between motivation and self-efficacy but not interest. The second approach considers a network of the total summed scores of interests, motivation, and self-efficacy for each specific SDG. Following this perspective, the centrality of nodes enclosed in the network can be identified. Addressing the most central nodes, network analysis highlights the most connected and thus emergent properties of a network [45,46].

Thus, having this in mind, the aim of the present study is to analyze the SDGPI in university students applying the network approach. According to the SDGPI’s theoretical model [18], the network analysis was run via a two-fold approach. For both approaches, the mean scores obtained at each SDG, reflective of the self-perception, were carefully considered. In the first approach, mean scores were inspected to establish whether edges occurred in association with very high, high, medium, low, or very low scores. In the second approach, mean scores were inspected to observe levels of self-perception in most central nodes. University students were selected as participants since the UN positioned educational environments as the heart of the strategy to promote SDGs [47,48]. Furthermore, to the best of our knowledge, an in-depth assessment of students’ psychological self-perception of SDGs has not been run, even though recent evidence suggested that the younger generations are more likely to support SDGs [49].

## 2. Materials and Methods

### 2.1. Participants and Procedure 

The current study was run on 417 Italian students at the University of Florence. They were 73.9% females and 26.1% males with a mean age of 22.2 (SD = 3.02) years. Three-hundred and thirty-three (79.9%) attended a bachelor’s degree course whereas 83 (19.9%) attended a master’s degree course. Most of them were recruited from the following faculties: Psychology (*n* = 351; 84.2%), engineering (*n* = 11; 2.6%), law (*n* = 11; 2.6%), and economy (*n* = 8; 1.9%). The participants were invited to participate in the research voluntarily, and before filling out the questionnaires, they were informed about the general purposes of the study. Those who voluntarily agreed to participate provided informed consent and a privacy protection disclaimer according to Italian privacy laws (Law Decree DL-196/2003) and European Union General Data Protection Regulation (EU 2016/679).

### 2.2. Measurement

The Sustainable Development Goals Psychological Inventory (SDGPI)—Italian version [18] (SDGPI) is a self-report tool composed of 51 items grouped into three factors enclosing 17 items each, namely, interest, motivation, and self-efficacy. The 17 items are parallel to the 17 SDGs for each of the factors. The interest factor investigates participants’ interest in each SDG. The motivation factor measures motivation to take practical action toward achieving each of the SDGs. The self-efficacy factor assesses the people’s self-efficacy to take practical action toward achieving each of the SDGs. The participants were asked to rank interest, motivation, and self-efficacy on a 5-point Likert scale ranging from 1 (not at all) to 5 (very much). The Italian scale showed satisfactory psychometric properties with good internal consistency coefficients of all the subscales (interest, α = 0.91; motivation, α = 0.92; self-efficacy, α = 0.94).

### 2.3. Data Analysis

Data analysis was conducted via the RStudio for Macintosh (RStudio BPC, Boston, MA, USA; Version 1.3.959). Network analyses were performed following the reporting standards for psychological network analyses in cross-sectional data [50]. Following the study’s aims, we applied network analyses in two steps. First, we ran a network analysis on the 51 items of the SDGPI, and we investigated the strongest edges among interest, motivation, and self-efficacy related to each of the 17 SDGs. Second, we conducted a network analysis on the sum of interest, motivation, and self-efficacy for each of the SDGs to detect the most central ones among university students participating in this study. The R packages bootnet 1.5 [51], mgm 1.2-9 [52], qgraph 1.9.2 [53], and mokken 3.0.6 [54] were used.

#### 2.3.1. Network Analysis on Interest, Motivation, and Self-Efficacy

In accordance with Epskamp and Fried [46], we inspected the network structure of the 51 items of SDGPI via an EBIC graphical LASSO network estimation. In the EBIC–LASSO model of SDGPI, nodes reflect the 51 items and edges indicate regularized partial correlations between nodes (blue = positive association; red = negative association; thicker edges = highly saturated) [46]. Moreover, in the EBIC–LASSO model of the SGDPI, each SDG has one item assessing interest, one item assessing motivation, and one item assessing self-efficacy. Thus, we examined the network structure of the model, taking into account the strongest and statistically significant edges across the same SDG, checking five occurrences: First occurrence: Strong and statistically significant edges between interest, motivation, and self-efficacy; second occurrence: Strong and statistically significant edges between interest and motivation but not self-efficacy; third occurrence: Strong and statistically significant edges between interest and self-efficacy but not motivation; fourth occurrence: Strong and statistically significant edges between motivation and self-efficacy but not interest; fifth occurrence: No statistically significant edges linking two dimensions. Moreover, we examined the level of overall self-perception associated with each occurrence concerning each SGD to determine whether the presence of edges co-occurred in association with high or low scores. Based on the overall mean score observed in our participants (M = 10.69; SD = 1.17), the SDG scores were ordered as follows: Very high > 13.03 (M + 2SD); high ≤ 13.03 (M + 2SD), ≥11.85 (M + 1SD); medium < 11.85 (M + 1SD), ≥9.52 (M − 1SD); low < 9.52 (M − 1SD), ≥8.35 (M − 2SD); very low < 8.35 (M − 2SD). 

#### 2.3.2. Network Analysis on Sum of Interest, Motivation, and Self-Efficacy

We inspected the network structure of the sum of interest, motivation, and self-efficacy for each of the 17 SDGs. Prior to running a network analysis, we checked via a Mokken scale analysis [55] whether the sum of interest, motivation, and self-efficacy for each SDG was a sufficient statistic. A Mokken coefficient of scalability (H_ij_) was considered to constitute a strong scale if 0.5 ≤ H_ij_ ≤ 1.0; a medium scale if 0.4 ≤ H_ij_ < 0.5; an acceptable scale if 0.3 ≤ H_ij_ < 0.4; and an unacceptable scale if H_ij_ < 0.3 [55]. Thereafter, we performed the EBIC graphical LASSO network estimations of the sum of interest, motivation, and self-efficacy for each SDG. In the EBIC–LASSO model, nodes reflect the 17 SDGs, and edges indicate regularized partial correlations between nodes (blue = positive association; red = negative association; thicker edges = highly saturated). The local network properties of the network were inspected via strength (a centrality index reflecting the sum of absolute weights of edges that connect a node to all other nodes) [56] and node predictability (the degree to which nodes are accounted for by other neighboring nodes) [57]. The robustness of the strength was inspected through strength stability and a bootstrapped difference test [45]. Strength was considered stable with a correlation stability (CS) coefficient of >0.50 [45]. The bootstrapped difference test for strength (2000 bootstraps; 95% CIs) calculates the statistically significant difference between the strengths of each node (central boxes = row value of strength; black boxes = statistically significant differences; gray boxes = no statistically significant differences) [45].

## 3. Results

**Figure 1:**
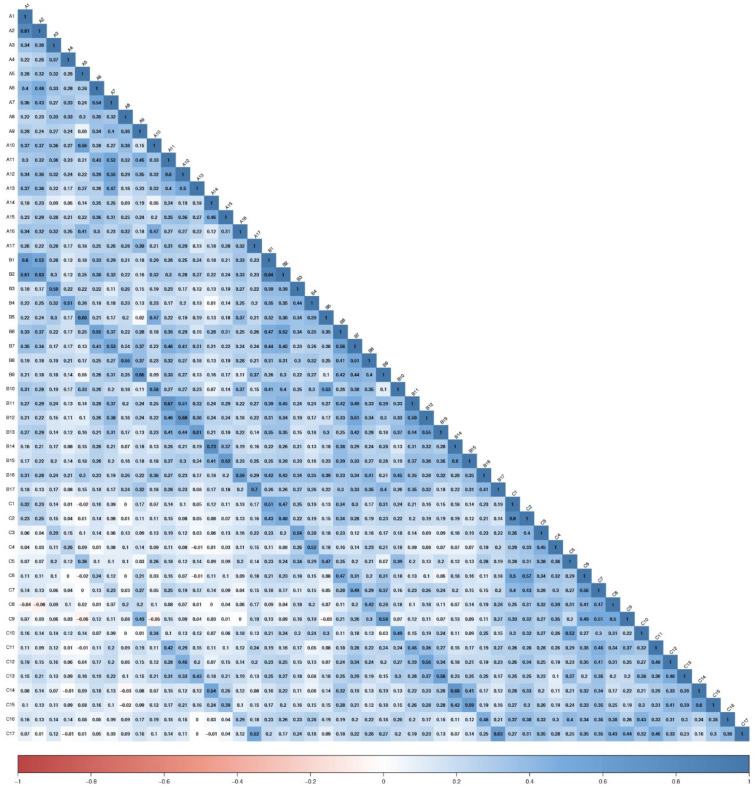
The Sustainable Development Goals Psychological Inventory: Zero-order polychoric correlations among scale items (*n* = 417).

**Figure 2:**
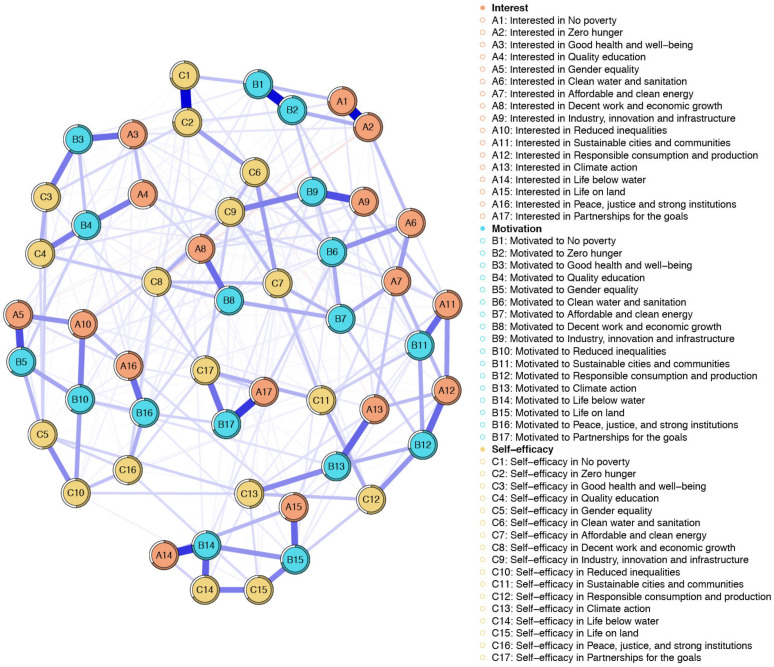
The Sustainable Development Goals Psychological Inventory: Graphical representation of the network model (*n* = 417).

**Figure 3:**
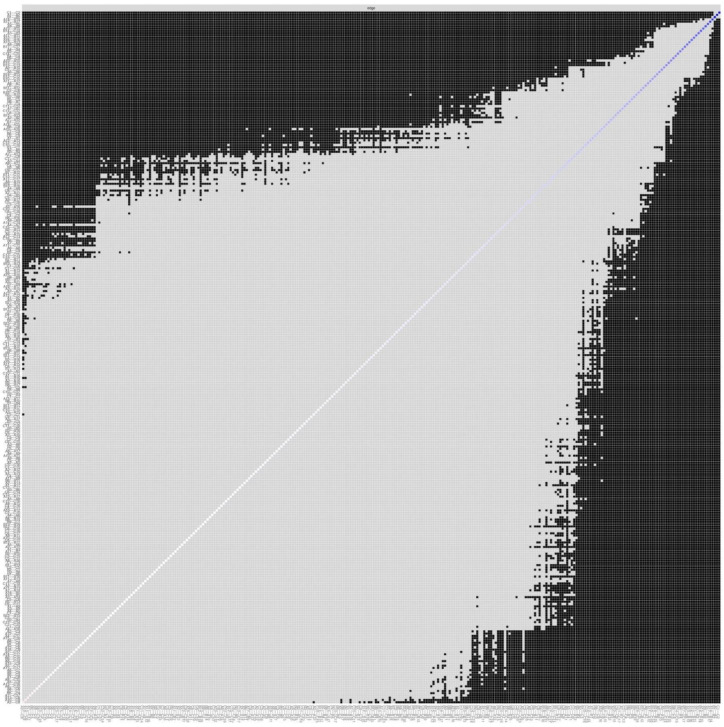
The Sustainable Development Goals Psychological Inventory: Difference test for the edge weight (*n* = 417). Note: X- and Y-axes represent, from lowest to highest values, all non-zero edges in the network. Statistical difference between two edge weights is represented with a black box; the non-statistical difference is indicated with a grey box. Labels of the two nodes connected by one edge are displayed at the side of each box. Each number reflects the number of SDGPl items.

Polychoric correlations of the 51 items of SDGPI are shown in Figure 1. Figure 2 shows the inspection of the local network structure and Figure 3 reports the non-parametric bootstrapped difference test for edge weight. Table 1 shows a summary of the visual inspection of the network structure and results of the non-parametric bootstrapped difference test for edge weight. Five SDGs fall into the category of the first occurrence, showing statistically significant and stronger edges between interest, motivation, and self-efficacy in the same SDG. Two SDGs showed high scores (Table 2). They were SDG 4 (Quality education) and SGD 13 (Climate Action). One SDG showed a medium score, namely SGD 15 (Life on land). Two SDGs showed low scores: SGD 14 (Life below water) and SGD 9 (Industry, innovation and infrastructure). Table 1 also shows that seven SDGs fall into the category of the second occurrence, reporting strong and statistically significant edges between interest and motivation but not self-efficacy in the same SDG. Among them, two SDGs were found with high scores and they were SDG3 (Good health and well-being) and SDG 5 (Gender Equality). Four SDGs displayed medium scores, and they were SDG 2 (Zero hunger), SDG 16 (Peace, justice, and strong institutions), SDG 11 (Sustainable cities and communities), and SDG 12 (Responsible consumption and production). One SDG was found to have a low score, namely SDG 1 (No poverty) (Table 1 and Table 2). Lastly, Table 1 reports that five SDGs belonged in the fifth occurrence, highlighting no statistically significant edges linking two or more dimensions in the same SDG. In the SDGs that were found in the fifth occurrence, one SDG had a high score, namely SDG 10 (Reduced inequalities). Three SDGs showed a medium score, and they were SDG 6 (Clean water and sanitation), SDG 7 (Affordable and clean energy), and SDG 8 (Decent work and economic growth). One SDG showed a low score, and it was SDG 17 (Partnerships for the goals) (Table 2). No SDGs were found in the third occurrence (strong and statistically significant edges between interest and self-efficacy but not motivation) nor in the fourth occurrence (strong and statistically significant edges between motivation and self-efficacy but not interest) (Table 1).

**Figure 4:**
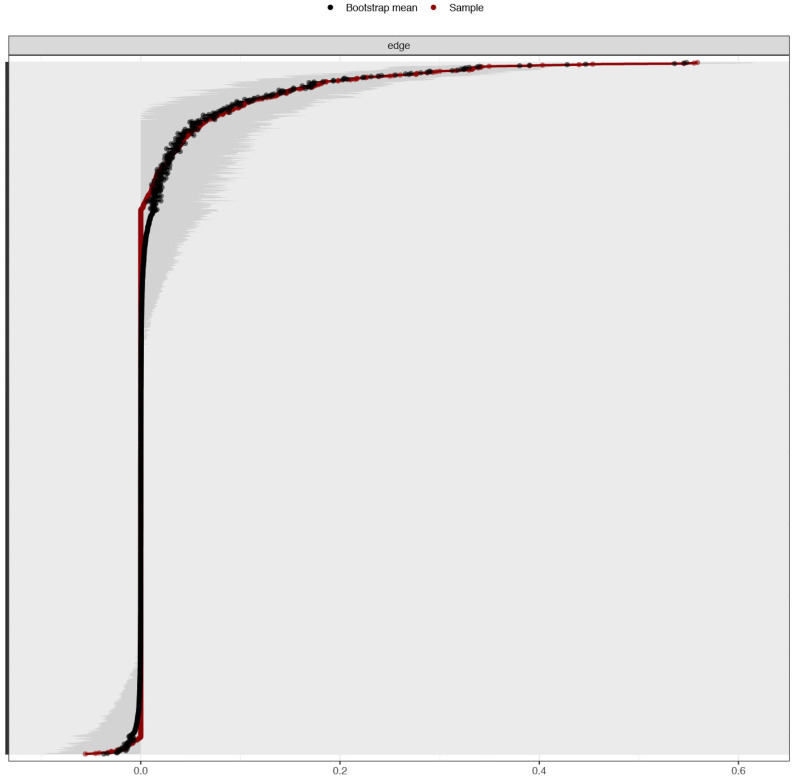
The Sustainable Development Goals Psychological Inventory: Bootstrap tests of the edge weight accuracy [95% confidence intervals] (*n* = 417). Red line shows the edge weight bootstrap and indicates values of the edges. The grey area represents 95% CIs. Each horizontal line displays an edge between two nodes ranked from the lowest to the highest edge weight. Each number on labels reflects a number of SDGPI items (i.e., node).

Figure 4 shows bootstrap tests of the edge weight accuracy, indicating good precision for all the edges of the network.

**Figure 5:**
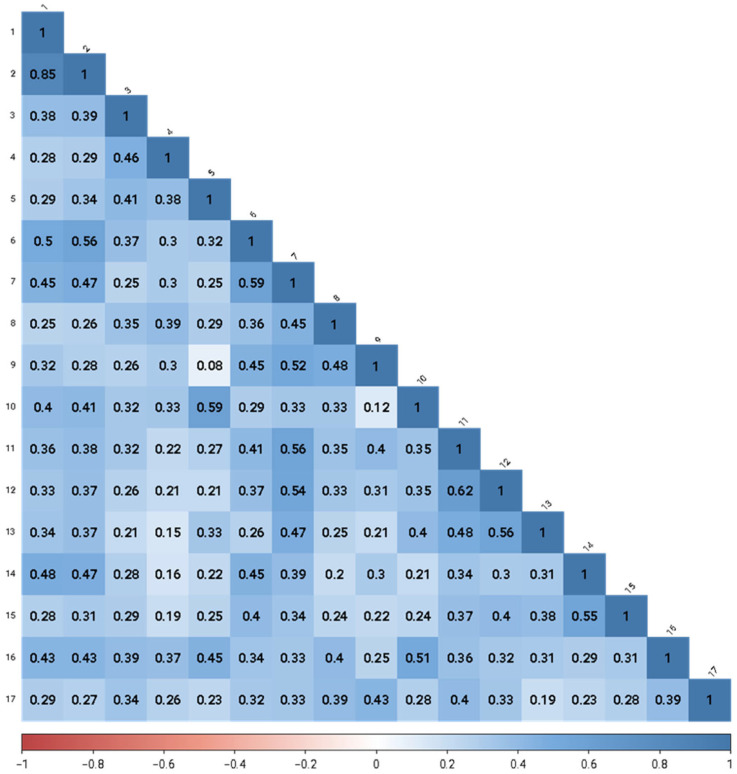
The Sustainable Development Goals Psychological Inventory, sum of interest, motivation, and self-efficacy for each Goal: Zero-order polychoric correlations among scale items (*n* = 417).

**Figure 6:**
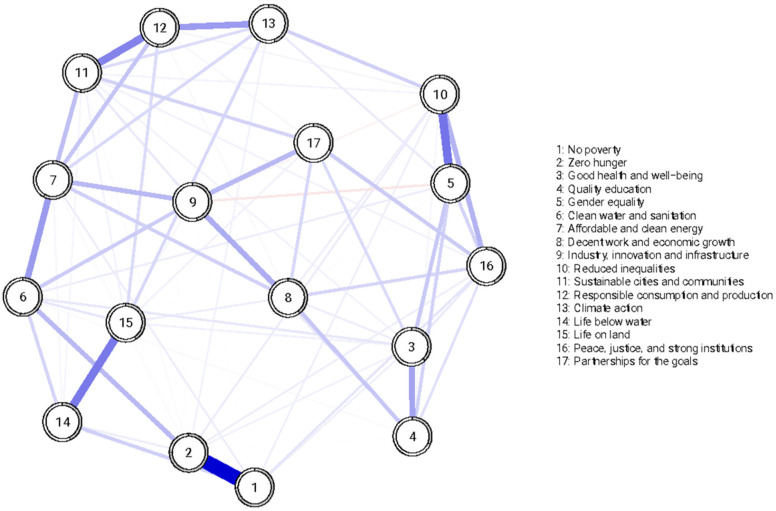
The Sustainable Development Goals Psychological Inventory, sum of interest, motivation, and self-efficacy for each Goal: Graphical representation of the network model (*n* = 417).

**Figure 7:**
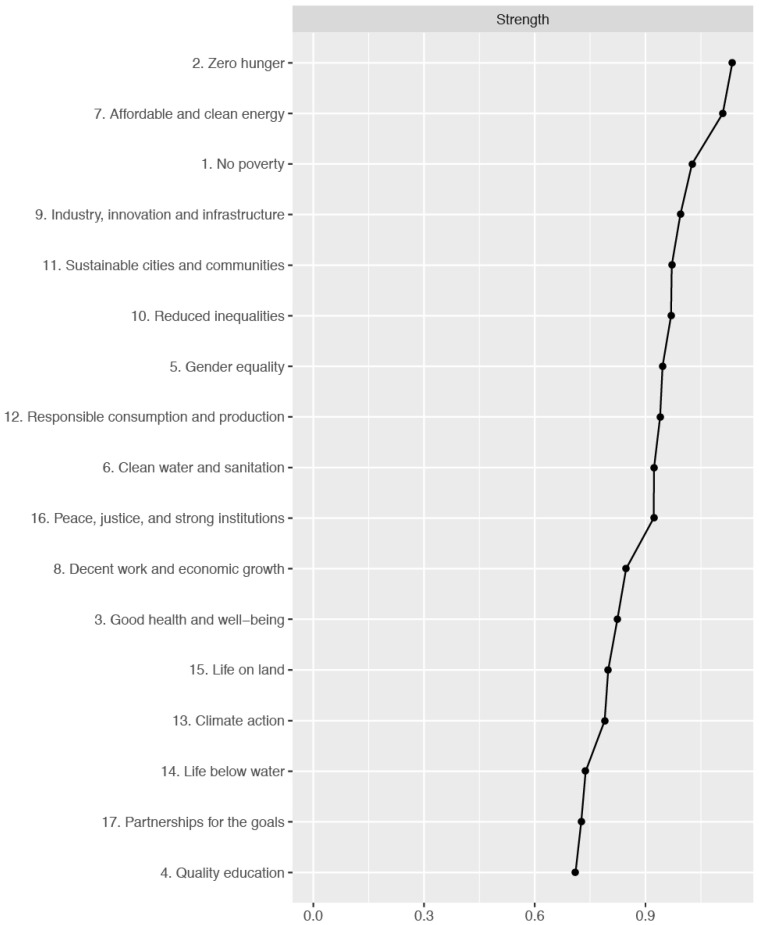
The Sustainable Development Goals Psychological Inventory, sum of interest, motivation, and self-efficacy for each Goal: Strength centrality estimates (*n* = 417).

**Figure 8:**
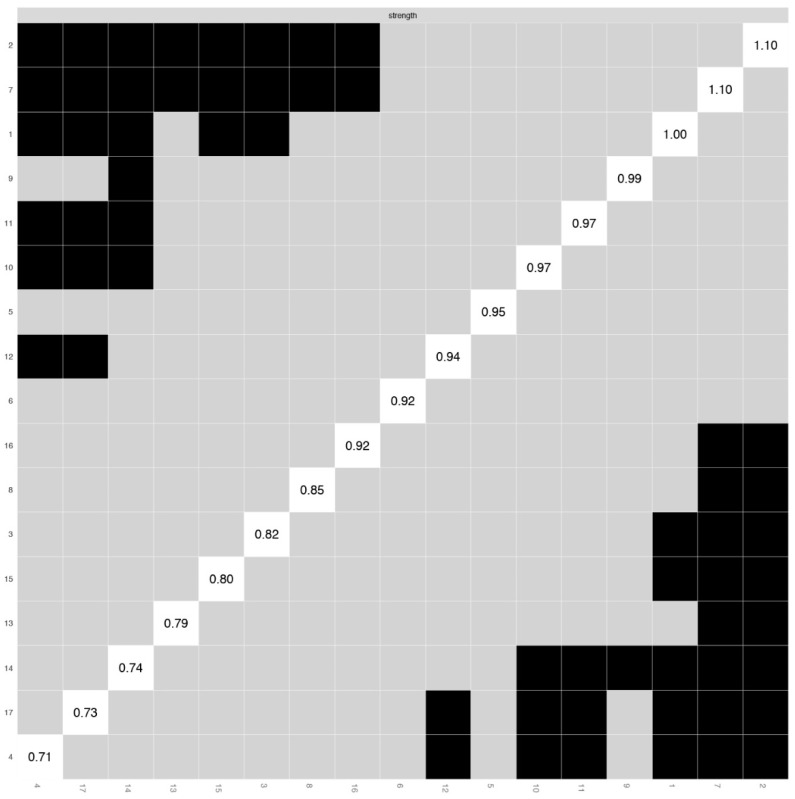
The Sustainable Development Goals Psychological Inventory, sum of interest, motivation, and self-efficacy for each Goal: Nonparametric bootstrapped difference test for strength (*n* = 417). Note: X- and Y-axes show, from lowest to highest value, all expected strength estimates for the WAMI items. The black box represents the statistical difference between two centrality estimates; and the grey box represents a non-statistical difference. White boxes show raw strength scores.

Table 1 displays item-level descriptive statistics for the sums of interest, motivation, and self-efficacy for each SDG (i.e., mean, standard deviation, skewness, kurtosis). Figure 5 reports polychoric correlations of the sums of interest, motivation, and self-efficacy for each SDG. Furthermore, Table 1 illustrates the results of the Mokken scale analysis. The sums of interest, motivation, and self-efficacy for each SDG showed good Mokken coefficients of scalability ranging from medium (Decent work and economic growth [H_ij_ = 0.46]) to strong (Life below water [H_ij_ = 0.70]. Figure 6, Figure 7 and Figure 8 show the inspection of the local network structure, revealing that two SDGs had the highest centrality index (strength) and were statically more central than the other nodes. They were SDG 2 (Zero Hunger; Strength = 1.10) and SDG 7 (Affordable and clean energy) (Strength = 1.10) (Figure 7 and Figure 8). Concerning the node predictability (Figure 6) of the sums of interest, motivation, and self-efficacy of SDGs, it ranges from 0.79 (node 2) to 0.32 (node 4) with mean node predictability of 0.42, showing that, on average, 42% of nodes’ variance can potentially be accounted for by the neighboring nodes. Furthermore, SDG 2 (Zero Hunger) showed the highest node predictability corroborating the evidence of its centrality in the network (Figure 6). Regarding the robustness of the network, the strength for the sums of interest, motivation, and self-efficacy of SDGs was high, indicating a trustworthy strength (CS coefficient = 0.64). Considering the score associated with the two most central nodes, both SDG 2 (Zero Hunger) and SDG 7 (Affordable and clean energy) showed medium scores.

## 4. Discussion

According to our knowledge, the present research was the first to implement a network analysis to study the SGDPI [18]. We performed a network analysis to expand our knowledge on the psychological self-perception of SDGs in our participants. Findings from network analysis expand those yielded via the factorial approach [18] by providing the SDGPI as a network composed of its constituent elements (items) [44]. In this framework, the first network analysis was run to inspect the different paths that connect interest, motivation, and self-efficacy for each SDG. The second network analysis was run to determine the most central SDGs in a network that parallels the sum of interest, motivation, and self-efficacy for each SDG.

Four main findings need to be emphasized and discussed. The first result concerns the SDGs with statistically significant edges linking interest, motivation, and self-efficacy in the same SDG (first occurrence). The second result deals with SDGs showing statistically significantly strong edges between interest and motivation but not self-efficacy in the same SDG (second occurrence). The third result is inherent to the SDG that displays no statistically significant edges linking two or more dimensions in the same SDG (fifth occurrence). The fourth result concerns the most central SDGs identified via the network analysis of the sum of interests, motivation, and self-efficacy.

According to the network theory, SDGs within the first occurrence (Table 3) could be interpreted as those SDGs in which the reciprocal interactions between interest, motivation, and self-efficacy in the same SDG could easily facilitate a sharper increase in self-perception via reciprocal reinforcement processes [45]. In those SDGs that already had high levels (SDG 4, Quality education; SDG 13, Climate action), the reciprocal reinforcement processes could facilitate them to remain high. Differently, those SDGs that were found ranging from medium (SGD 15, Life on land; SDG 14, Life below water) to low (SDG 9, Industry, innovation and infrastructure) show a progressive lack of self-perception. High scores observed in SDG 4 (Quality education) and SGD 13 (Climate Action) are in line with previous qualitative findings shown by Di Fabio and Rosen [18], which reported SDG 4 as the most important SDG and SDG 13 as the fourth most important SDG. Similarly, the medium levels observed for SGD 15 (Life on land) are consistent with past qualitative findings that showed SDG 15 neither in those SDGs that are the most important nor in those SDGs that are the less important [18]. Lastly, the low score reported by SGD 14 (Life below water) is in accordance with past qualitative data that highlighted SGD 14 as the least important SDG [18]. Consistently, the low score detected for SDG 9 (Industry, innovation and infrastructure) is in line with previous qualitative data, showing SDG 9 among the less important SDGs [18]. With that said, our results yielded via the network analysis build upon previous findings indicating that these five SDGs are connected by strong edges, thus reflecting an alignment of scores of interest, motivation, and self-efficacy (first occurrence). Furthermore, the presence of the first occurrence identifies those SDGs that could be facilitated by an increase via reciprocal reinforcement processes, highlighting a virtuous circle between interest, motivation, and self-efficacy. This is in line with the theoretical background of the SDGPI [18], as well as theories that have linked together interest, motivation, and self-efficacy, such as the Self-Determination Theory (SDT) [20] and the interests model [19].

Our results showed that the majority of SDGs (seven out of seventeen) fell in the second occurrence (Table 3), displaying a link in the SDGs between interest and motivation but not self-efficacy. This finding is in line with the previous quantitative data, which observed self-efficacy as the dimension with the lowest scores [18]. On one hand, our results indicated the lack of a link with self-efficacy in seven SDGs, but on the other hand, our findings also showed the presence of an edge between interest and motivation in these latter SDGs. In line with network theory [45], the presence of edges is reflective of a reciprocal reinforcement process between interest and motivation. This presence, according to the psychology of sustainability and sustainable development [14], could lead to a sharper increase in the zone of proximal development for self-efficacy [12,13]. The higher the score associated with an SDG, the greater the readiness with which the participants in this study could progress in the zone of proximal development for self-efficacy. Thus, among SDGs in the second occurrence, those with high scores could have high readiness to develop a zone of proximal development for self-efficacy, SDGs with medium scores could have medium readiness to develop a zone of proximal development for self-efficacy, and SDGs with a low score could have low but present readiness to develop a zone of proximal development for self-efficacy. Concerning the scores associated with SDGs in the second occurrence, two findings are different from previous qualitative data and need to be discussed. First, SDG 5 (Gender equality) was found to be high, whereas Di Fabio and Rosen [18] assessed it as one of the less important. This finding could be explained by the fact that our sample is composed of a higher percentage of female university students that were found to have a better concept of gender equality than males [58]. Furthermore, university students were also found to be highly favorable to recognizing the importance of reducing sexism, developing gender competencies, and developing a gender-sensitive pedagogy [59]. Second, SDG 1 (No poverty) was found to be with a low score, whereas Di Fabio and Rosen [18] identified it as one of the most important. Again, it could be explained by the fact that our sample is composed of university students from a highly developed zone, in which the rate of poverty is among the lowest compared with other regions of Italy [60]. As a consequence, our participants may be not interested or motivated and have low self-efficacy concerning an SDG that is not directly experienced in their psychosocial environment. Furthermore, most recent data on poverty in Italy showed that poverty was negatively associated with educational level, showing the lowest level among people with a high-school degree or more [60].

On the basis of our results, five SDGs were in the fifth occurrence (Table 3), showing a lack of edges connecting interest, motivation, and self-efficacy. According to the network realm, this occurrence did not provide these SDGs with the reciprocal reinforcement processes fostered by the presence of edges [45]. The absence of edges is also reflective of a high absence of alignment among scores of interest, motivation, and self-efficacy, showing the lack of relevant association in the network model. Among these SDGs, SDG 10 (Reduced inequalities) showed a high score, in line with Di Fabio and Rosen [18] who found SDG 10 among the most important SDGs. Similarly, medium scores observed for SDG 6 (Clean water and sanitation), SDG 7 (Affordable and clean energy), and SDG 8 (Decent work and economic growth) are in line with the findings of Di Fabio and Rosen [18]. Lastly, the low score associated with SDG 17 (Partnerships for the goals) is also in line with Di Fabio and Rosen [18] who highlighted that SDG 17 was the second least important. However, concerning the five SDGs in the fifth occurrence, the network analysis suggested an absence of connection between interest, motivation, and self-efficacy, highlighting the deep need to activate interactions between the three dimensions that seem to not display any connection. It should be noted that no SDGs showed edges linking interest and self-efficacy but not motivation, nor edges among motivation and self-efficacy but not interest. It is in line with the theoretical framework of the SDGPI [18], which conceives the path to action from interest to motivation and self-efficacy and their virtuous circle.

Our results showed that the network of summed scores of interests, motivation, and self-efficacy revealed the two most central SDGs. They were SDG 2 (Zero Hunger) and SDG 7 (Affordable and clean energy), having medium levels, in line with Di Fabio and Rosen’s [18] findings. Network analysis revealed that these two SDGs shared the highest number of connections with all other surrounding nodes. It indicates that an increase in the levels of self-perception in these SDGs could modify the levels of the other neighboring nodes. Thus, most central SDGs are also those that can facilitate the modification of the self-perception of other SDGs. This is in line with the SDGs as conceived in the 2030 Agenda, which has indivisibility and interdependence as core features [6,7]. 

The current research has strengths and limitations. The main strength is that the network approach has been applied for the first time to the SDGPI, consistently with its theoretical framework, assessing interest, motivation, and self-efficacy in relation to the 17 SDGs to provide tailor-made actions. Our results expand the previous qualitative data on the SDGPI [18] advancing the network realm as a promising tool to analyze the psychological self-perception of SDGs as a network of interacting items. In this view, applying network analysis has shown results also able to provide a promising direction for tailor-made interventions. According to our results, researchers and practitioners could consider tailor-made interventions, taking into account the overall self-perception detected in each SDG and also the occurrence observed for each SDG. The SDGs that fell in the first occurrence have the advantage of enclosing the reciprocal interaction process between interest, motivation, and self-efficacy, making it possible to achieve a sharper increase in the levels of self-perception through the intervention. In this line, when addressing the SDGs that fell in the first occurrence, on one hand, the interventions could be promising in fostering these SDGs at a reasonable pace with a higher likelihood of favorable outcomes. On the other hand, the interventions have to be tailored progressively based on the level of scores, considering that low scores indicate greater difficulties and the need to progress, while high scores suggest the need to reinforce the positive self-perception that emerged and disambiguate from the risk of social desirability. The SDGs that fell in the second occurrence have the advantage of enclosing a reciprocal interaction process between interest and motivation, making it possible to activate a zone of proximal development for self-efficacy through tailor-made interventions, considering that more suitable SDGs could be those in the second occurrence and high or medium scores, and the SDGs with low scores could be the most problematic. The SDGs that fell in the fifth occurrence are those that require more effort to be increased and to achieve a trustworthy psychological self-perception of the SDGs through interventions. Lastly, the most central SDGs could be promising for interventions that aim to have an effect on increasing the overall self-perception of SDGs [24], fostering a general self-perception in the framework of the seventeen sustainable goals. 

The main limitation of the current study is its cross-sectional design. Thus, we cannot infer whether a given node causes or is caused by another linked node. Consequently, future research could investigate the networks of SDGPI by also implementing a longitudinal analysis of interest, motivation, and self-efficacy. Furthermore, our study enrolled only students from a specific area of Italy and most of them attended courses in psychology. Thus, our results cannot be extended to Italian university students. Future studies should investigate the SDGPI network, with participants from universities located in different zones of Italy Furthermore, the network analysis of the SDGPI could be also carried out in other countries to compare the obtained results. Moreover, since the participants in the present study are only university students, further research on the SDGPI network could also be conducted considering different targets, such as high-school students, workers including vulnerable and precarious workers [61,62], retired people, and the unemployed. Thus, although future research is needed, the network perspective is promising in identifying those particular SGDs to be addressed in particular targets, contexts, areas, and countries for planning tailor-made interventions directed towards enhancing the objectives of the 17 SGDs. Network analysis could provide researchers with more accurate information concerning the participants assessed via the SGDPI. For example, if practitioners are interested in increasing the global self-perception of SDGs, they could plan actions to address the most central SDGs. Differently, if practitioners are interested in stimulating the self-efficacy associated with SDGs, they could focus on those SDGs that fell in the second occurrence, taking advantage of the zone of proximal development for self-efficacy. In another case, when temporal or material resources have less availability, practitioners could address SDGs that fell in the first occurrence, using the possibility of a sharper increase in self-perception. Overall, as noted above, interventions could be gradually tailored based on the level of the SDG scores (e.g., from the highest to the lowest).

## 5. Conclusions

Our study showed the possibility of bringing out different levels of self-perception in participants and different criticalities in reference to the self-perception of the 17 SGDs. In this framework, it seems highly important to increase the culture of prevention by articulating it in primary, secondary, and tertiary preventive actions, with reference to the 17 SGDs and their links between interest, motivation, and self-efficacy. This latter awareness of links could reverberate in the concreteness of different and specific tailor-made interventions to enhance positive behaviors, paying attention to specificities of targets and contexts, promoting specific actions anchored to the results of empirical research [18], and supporting principles of accountability. In the current period of immense challenges and changes, as well as the economic crisis [63,64], the principles of accountability are increasingly relevant to provide effective interventions without wasting the limited available resources. Accountability principles [65,66,67] show concern for service costs, intervention effectiveness, and best practices supported by research [68]. This study supports the relevance of SDGPI in enriching the sustainability framework with new awareness and suggestions for tailor-made positive strength-based preventive actions [17] for primary preventive actions [12,13,26,27,66,69].

## Figures and Tables

**Table 1:** Summary of occurrences of statistically significant edges between interest, motivation, and self-efficacy.

Sustainable Development Goals	1st Occurrence	2nd Occurrence	3rd Occurrence	4th Occurrence	5th Occurrence
1. No poverty		✓			
2. Zero hunger		✓			
3. Good health and well-being		✓			
4. Quality education	✓				
5. Gender equality		✓			
6. Clean water and sanitation					✓
7. Affordable and clean energy					✓
8. Decent work and economic growth					✓
9. Industry, innovation and infrastructure	✓				
10. Reduced inequalities					✓
11. Sustainable cities and communities		✓			
12. Responsible consumption and production		✓			
13. Climate action	✓				
14. Life below water	✓				
15. Life on land	✓				
16. Peace, justice, and strong institutions		✓			
17. Partnerships for the goals					✓

Note: First occurrence: Strong and statistically significant edges between interest, motivation, and self-efficacy in the same SDG; second occurrence: Strong and statistically significant edges between interest and motivation but not self-efficacy in the same SDG; third occurrence: Strong and statistically significant edges between interest and self-efficacy but not motivation in the same SDG; fourth occurrence: Strong and statistically significant edges between motivation and self-efficacy but not interest in the same SDG; fifth occurrence: No statistically significant edges linking two or more dimensions in the same SDG.

**Table 2:** The Sustainable Development Goals Psychological Inventory, sum of interest, motivation, and self-efficacy for each Goal: Means, standard deviations, skewness, and kurtosis (*n* = 417).

Sustainable Development Goals	Mean	Sd	Min	Max	Skewness	Kurtosis	H_ij_(SE)	Score Evaluation
1. No poverty	9.90	2.37	3	15	−0.07	−0.30	0.55 (0.03)	Low
2. Zero hunger	10.10	2.37	3	15	−0.18	−0.14	0.52 (0.03)	Medium
3. Good health and well-being	12.00	2.11	4	15	−0.78	0.61	0.55 (0.04)	High
4. Quality education	11.63	2.15	4	15	−0.52	0.19	0.50 (0.04)	High
5. Gender equality	12.65	2.18	3	15	−1.19	1.62	0.58 (0.04)	High
6. Clean water and sanitation	10.35	2.40	3	15	−0.16	−0.29	0.46 (0.03)	Medium
7. Affordable and clean energy	10.51	2.31	3	15	−0.21	−0.11	0.47 (0.04)	Medium
8. Decent work and economic growth	11.05	2.16	4	15	−0.32	−0.22	0.44 (0.04)	Medium
9. Industry, innovation and infrastructure	8.72	2.70	3	15	0.07	−0.48	0.62 (0.03)	Low
10. Reduced inequalities	12.27	2.25	4	15	−0.79	0.21	0.54 (0.04)	High
11. Sustainable cities and communities	10.44	2.51	3	15	−0.37	−0.10	0.57 (0.03)	Medium
12. Responsible consumption and production	10.86	2.62	3	15	−0.41	−0.16	0.63 (0.03)	Medium
13. Climate action	11.48	2.55	3	15	−0.68	0.26	0.60 (0.03)	High
14. Life below water	8.67	2.73	3	15	−0.01	−0.74	0.70 (0.03)	Low
15. Life on land	10.56	2.57	3	15	−0.41	−0.03	0.60 (0.03)	Medium
16. Peace, justice, and strong institutions	11.47	2.35	3	15	−0.56	0.21	0.51 (0.04)	Medium
17. Partnerships for the goals	9.13	2.93	3	15	−0.02	−0.54	0.67 (0.03)	Low

Note: H_ij_ = Mokken Coefficient of scalability. Very high > 13.03 (M + 2SD); high ≤ 13.03 (M + 2SD), ≥11.85 (M + 1SD); medium < 11.85, (M + 1SD), ≥9.52 (M − 1SD); low < 9.52 (M − 1SD), ≥8.35 (M − 2SD); very low < 8.35 (M − 2SD).

**Table 3:** Overall interpretation of results yielded via network analysis and score evaluation of the SDGPI.

Sustainable Development Goals (SDGs)	Occurrence	Score Evaluation	Interpretation
4. Quality education	1st	High	Reciprocal reinforcement processes + high self-perception of SDG
13. Climate action	1st	High	Reciprocal reinforcement processes + high self-perception of SDG
15. Life on land	1st	Medium	Reciprocal reinforcement processes + medium self-perception of SDG
14. Life below water	1st	Low	Reciprocal reinforcement processes + low self-perception of SDG
9. Industry, innovation and infrastructure	1st	Low	Reciprocal reinforcement processes + low self-perception of SDG
3. Good health and well-being	2nd	High	Zone of proximal development for self-efficacy + high self-perception of SDG
5. Gender Equality	2nd	High	Zone of proximal development for self-efficacy + high self-perception of SDG
2. Zero hunger	2nd	Medium	Zone of proximal development for self-efficacy + medium self-perception of SDG
16. Peace, justice, and strong institutions	2nd	Medium	Zone of proximal development for self-efficacy + medium self-perception of SDG
11. Sustainable cities and communities	2nd	Medium	Zone of proximal development for self-efficacy + medium self-perception of SDG
12. Responsible consumption and production	2nd	Medium	Zone of proximal development for self-efficacy + medium self-perception of SDG
1. No poverty	2nd	Low	Zone of proximal development for self-efficacy + low self-perception of SDG
10. Reduced inequalities	5th	High	No reciprocal reinforcement processes + high self-perception of SDG
6. Clean water and sanitation	5th	Medium	No reciprocal reinforcement processes + medium self-perception of SDG
7. Affordable and clean energy	5th	Medium	No reciprocal reinforcement processes + medium self-perception of SDG
8. Decent work and economic growth	5th	Medium	No reciprocal reinforcement processes + medium self-perception of SDG
17. Partnerships for the goals	5th	Low	No reciprocal reinforcement processes + low self-perception of SDG

Note: First occurrence: Strong and statistically significant edges (alignment) between interest, motivation, and self-efficacy; second occurrence: Strong and statistically significant edges between interest and motivation but not self-efficacy in the same SDG; fifth occurrence: No statistically significant edges linking two dimensions (no alignment). Third and fourth occurrences were not found (strong and statistically significant edges between interest and self-efficacy but not motivation; strong and statistically significant edges between motivation and self-efficacy but not interest, respectively).

## Data Availability

The data presented in this study are available on reasonable request from the corresponding author. The data are not publicly available due to privacy reasons.
